# The impact of rehabilitation on the community life of stroke survivors in Accra, Ghana

**DOI:** 10.4102/sajp.v79i1.1839

**Published:** 2023-02-28

**Authors:** Tawagidu Mohammed, Gifty G. Nyante, Joyce D. Mothabeng

**Affiliations:** 1 Department of Physiotherapy, School of Healthcare Sciences, University of Pretoria, Pretoria, South Africa; 2 Department of Physiotherapy, School of Biomedical and Allied Health Sciences, University of Ghana, Accra, Ghana

**Keywords:** stroke, stroke survivors, rehabilitation, community life, Ghana

## Abstract

**Background:**

Return to pre-stroke life is of great importance to stroke survivors, their families and communities as stroke affects their ability to perform activities of daily living. It is therefore important to understand the impact of stroke rehabilitation on the community life of stroke survivors in Ghana as there are limited data.

**Objectives:**

Our study aimed to explore and describe the views of stroke survivors on the impact of stroke rehabilitation on their community life.

**Method:**

A descriptive qualitative study was conducted among 15 stroke survivors recruited from three selected hospitals in the Greater Accra Region of Ghana. Individual in-depth interviews were conducted using a semi-structured interview guide. Interview transcripts were analysed using thematic analysis and this gave rise to several themes.

**Results:**

The authors found that stroke left most of the survivors with functional limitations and they required various degrees of assistance to perform their activities of daily living. As the stroke survivors received rehabilitation, most of them mentioned improvements in function. However, most participants were still unable to return to work and enjoy social or leisure activities.

**Conclusion:**

Our study shows that attention needs to be given to the occupational and social management in rehabilitation as much as it is given to the physical management, to improve community integration post-stroke.

**Clinical implications:**

Our study highlights the need to take into consideration the occupational and social aspects of life as part of the rehabilitation process for stroke survivors.

## Introduction

Stroke, which is a debilitating disorder, still remains a major public health concern worldwide (Moskowitz, Lo & Iadecola 2010). Although there has been much improvement with stroke care, stroke survivors still remain with functional deficits (Baatiema et al. 2017). Mild to severe disabilities that accompany stroke make survivors dependent on others for performance of their activities of daily living, which leads to poor quality of life (Badaru et al. 2017). In Ghana, about 48% of stroke survivors fall within a working age group. This, therefore, poses a huge economic burden on the individual, family and the nation (Owolabi et al. 2018) as this group of people form the working population in Ghana. It is therefore important for stroke survivors to reintegrate back into their communities after stroke, to perform their normal functional activities of daily living and thereby, have productive lives (Chinchai, Sirisatayawong & Jindakum 2020).

Stroke rehabilitation has been found to improve the stroke-related physical deficits or disability accompanying the condition and reintegrate stroke survivors back into their communities (Yagi et al. 2017). As the main aim of rehabilitation of stroke survivors is to get them back to their pre-stroke life as well as get them back to work and social life, assessing the impact of stroke rehabilitation on the community life of stroke survivors has become important. This will help clinicians to understand the extent to which stroke survivors are able to return to their pre-stroke life after stroke. Community life in our study takes into consideration the previous roles the stroke survivor assumed before the stroke. This includes the physical, social and economic roles of the survivor. Being able to reintegrate and assume the aforementioned roles is the ultimate goal of rehabilitation. Rehabilitation will be seen to be successful once these are achieved.

In Ghana, there are limited data on stroke rehabilitation. There is also limited information on the impact of stroke rehabilitation on the community life of stroke survivors in Ghana, especially from the stroke survivor’s point of view. It is, however, important to understand the impact of stroke rehabilitation on the community life of stroke survivors as it will help to understand the outcome of the rehabilitation given. Our study, therefore, aimed to explore and describe the views of stroke survivors on the impact of stroke rehabilitation on their community life.

## Methods

A descriptive qualitative study was conducted employing a phenomenological approach, to help understand the impact of stroke rehabilitation on the community life of stroke survivors. It was carried out at the outpatient physiotherapy units of three selected hospitals in the Greater Accra Region of Ghana. One hospital each was selected from each of the three levels of healthcare in Ghana. These are primary, secondary and tertiary hospitals. All the selected hospitals are involved in both inpatient and outpatient stroke rehabilitation.

### Participants and sampling strategies

Fifteen stroke survivors who were receiving outpatient physiotherapy rehabilitation were purposively sampled from the three selected hospitals, five from each. Participants were recruited if they had received outpatient physiotherapy rehabilitation for at least 6 months and were first-time stroke survivors. The authors excluded persons with recurrent stroke because of the residual disability with recurrent stroke. Purposive sampling is a non-probabilistic sampling procedure used in sampling participants in qualitative studies allowing researchers to choose the sample based on who they think best fits in a study (Campbell et al. 2020).

### Data collection

The first author visited the outpatient physiotherapy departments and identified stroke survivors who met the inclusion criteria. The purpose of our study was carefully explained to the participants and participation was completely the choice of the participants. Informed consent was sought and received from each sampled participant. Individual in-depth interviews were conducted using a semi-structured interview guide and the first author was the moderator of the interview. Individual in-depth interviews are optimal for collecting data on individuals’ personal histories, perspectives, and experiences, particularly when sensitive topics are being explored (Jamshed 2014). With this approach, respondents can situate their own life experience within a larger social context. Interviews were conducted at a mutually agreed time. Before the start of each interview, the interviewer (first author) engaged participants in informal conversation to establish rapport and prepare them for the interview. With the permission of each participant, the interviews were audio-taped using a digital voice recorder. During and at the end of the interview, the first author restated or summarised the participants’ contributions to confirm that their views had been accurately captured. The interviews focused on three main areas to help understand the impact of rehabilitation on the stroke survivors. Questions on pre-stroke life, life after stroke and life after 6 months of rehabilitation were discussed. The audio-taped interviews were transcribed to Microsoft Word for data analysis.

### Data analysis

Data analysis occurred concurrently with data collection to ensure data saturation. Interview transcripts were converted to Microsoft Word, imported and sorted into NVivo 11 software for data management. Thematic analysis of transcripts was performed, and the stages of thematic analysis served as a guide for the analysis process. This involved familiarising with the data, generating codes, searching for themes, reviewing themes, defining themes and then producing the report (Maguire & Delahunt 2017). Authors read and re-read the transcripts for familiarisation. Transcripts were assigned identification codes. A code book was also developed and themes were generated. The first two authors worked together to develop the codes. Themes and subthemes were then generated upon discussion among the authors. The third author cross-checked the coding and themes developed. There was member checking and bracketing to ensure the rigour required for data analysis.

## Results

### Outpatient rehabilitation for stroke survivors

The authors sampled first-time stroke survivors undergoing outpatient rehabilitation for at least 6 months. All participants mentioned receiving only outpatient physiotherapy rehabilitation post-discharge from acute care in addition to their medical reviews. Only one respondent reported also undergoing herbal treatment in addition to the outpatient physiotherapy management:

‘I do herbal treatment in addition to my management.’ (Participant 11, male, welder)

The results are presented based on the themes that emerged from the analysis.

### Theme 1: Life before stroke

All participants reported being very functional and independently carrying out their activities of daily living ranging from their personal or self-care activities, to work, to family or home roles and social or leisure activities. With respect to their selfcare before the stroke episode, they were capable of carrying out all their selfcare activities independently. They could bath, groom, feed, move around as well as carry out their marital roles independently. With regard to family or home roles, they reported carrying out these roles successfully. They interacted well with their families, helped with chores around the home, had quality time with children, and so forth. They could cook, clean, and perform their roles at home independently:

‘Then I was doing everything around the home, with no assistance.’ (Participant 10, male, clerk)

‘I was cooking and cleaning and doing everything by myself.’ (Participant 8, female, entrepreneur)

‘I used to help my children with their homework.’ (Participant 3, female, cleaner)

Almost all reported having some form of employment prior to the stroke episode. They were also able to carry out their work activities successfully without any assistance from others:

‘I carried out all the duties myself. Per the nature of the work, I lift items a lot. I sell crates, the crates that are used for mineral bottles and provision. And so, I do some form of lifting every now and then. I also do this whenever I choose.’ (Participant 14, male, welder)

‘I was very active at work; I don’t sit down at all.’ (Participant 9, male, footballer)

‘I was a driver. For the four and half years, I haven’t been on leave before. I work seven (7) days in a week.’ (Participant 13, male, driver)

Most also mentioned being socially active. They attended social and religious events regularly and also enjoyed some leisure activities prior to the stroke episode:

‘I used to go for funerals, parties, naming ceremonies an all, even church service I was going.’ (Participant 2, male, welder)

‘Yes, I do go for weddings, church activities.’ (Participant 12, female, entrepreneur)

‘I used to play football but currently it’s more of attending social events like funerals, parties and stepping out.’ (Participant 5, male, transport officer)

### Theme 2: Life after stroke

All participants spontaneously reported having various impairments and activity limitations when asked how stroke had affected their lives. They reported that stroke had affected their ability to function and carry out their activities of daily living. Most reported on their inability to perform basic self-care or personal activities such as bathing, grooming, feeding, among others independently:

‘After the stroke, I couldn’t wash. I couldn’t do basically so many things. So many things like … I couldn’t feed myself, so they used to feed me, then I’d be given my bath.’ (Participant 1, male, civil servant)

‘At the time I had stroke, I could not bath, I could not sit, I could not do anything.’ (Participant 2, male, laboratory technician)

‘I couldn’t do anything at all. I had to either feed myself or eat with my left hand and also my wife used to bath for me.’ (Participant 9, male, footballer)

They reported on their inability to carry out their family or home roles. Most of the participants also reported being bedridden and as such, they could not go to work or be involved in social or leisure activities as they could not walk:

‘Oh, I wasn’t able to do anything. I wasn’t able to wash my clothes. It even looked as if my hand was getting crooked.’ (Participant 12, female, entrepreneur)

‘I could not carry out normal household duties. I am assisted with a lot of them. With work, I was not able to go.’ (Participant 3, female, cleaner)

‘I couldn’t do anything anymore and I wasn’t able to go to work.’ (Participant 11, male, welder)

### Theme 3: Life after 6 months of rehabilitation

Following 6 months of rehabilitation, most participants mentioned having some improvement in function and they were able to perform some activities of daily living after the stroke. Most of them mentioned being able to perform most of their selfcare or personal activities such as bathing, grooming, continence, and feeding, independently although they had challenges with mobility, especially walking:

‘I cannot stand properly. I cannot stand on my leg.’ (Participant 6, female, baker)

‘Right now, what’s left is improving on my walking.’ (Participant 9, male, footballer)

Some mentioned being able to carry out their family or home duties such as cooking, cleaning, washing, taking care of family and children, and so forth, although not completely independently and sometimes needed some assistance. Some participants also mentioned that they could not carry out all their selfcare and home or family duties independently and needed some form of support because they do not have complete function of the affected hand:

‘Oh, I support. You see, when using just one hand, it’s challenging. But I try my best.’ (Participant 14, male, welder)

‘I do bath. I use one of the hands to take the sponge to bath, wash here wash my hand. Then I call my small boy to come and bath my back. And it is because of the hand that is not very functional.’ (Participant 7, male, oil refinery officer)

‘Right now, what’s left is improving on my steps and my hands.’ (Participant 9, male, footballer)

With regard to return to work post-stroke, almost all mentioned that they had been unable to return to work because of the nature of their work as they had not regained full function, although they were recovering and seeing improvement. Most were optimistic that they could return to work when recovery of function gets better:

‘I’ve not started work yet because sometimes I get tired after walking after a little while.’ (Participant 15, male, environmental officer)

‘It’s with going back to work. As for that one, it shouldn’t take too long for me to return back to work.’ (Participant 12, female, entrepreneur)

‘I can do everything. Except work that I cannot go. I’m a technician. I’m a laboratory technician. I take blood samples, and work on the blood. And print the results out. But now I cannot take samples, I cannot work, I cannot write. So that’s why I’m not going.’ (Participant 2, male, laboratory technician)

For social engagement or leisure, most participants mentioned they were unable to attend social gatherings or even enjoy leisure activities, although they had gained some functional improvement. Most acknowledged that they were not able to attend social functions or enjoy leisure because they were trying to stay away from people because of their condition and not because they were not doing well functionally. Some of them acknowledged they could walk but were left with residual disability. They think people will talk about them and also did not want to invite the stares of people, which makes them feel embarrassed about their situation:

‘I don’t feel like associating myself with other people.’ (Participant 15, male, environmental officer)

‘Since that time, I haven’t attended any occasion. When such occasions are being held, I will get out from the house. I am able to walk. When I go into the midst of people, I feel embarrassed. I feel like people are staring at me.’ (Participant 3, female, cleaner)

‘I don’t go out at all. It is frustrating for me. When they see you, they will ask what happened to you. Then you feel sorrowful.’ (Participant 6, female, baker)

‘We don’t go for parties; we don’t go to church too much like we used to. We don’t go to the beach too. Because people will be watching you, saying all manner of things; so, you kind of feel shy small and that makes you coil in.’ (Participant 10, male, clerk)

Overall, participants acknowledged the importance of rehabilitation as it had helped them in their journey to functional recovery. Most of them also mentioned that they see themselves doing better in the next few months to a year if they adhered to their rehabilitation schedules:

‘As you can see, I’m recovering by God’s grace. So, I see myself to have improved a lot than I was before this moment. Physiotherapy has really been helpful, because, I couldn’t do certain things on my own previously but after some sessions, I can see a great improvement in my health. I can see progress.’ (Participant 8, female, entrepreneur)

‘It’s going to be massive. Initially, whenever I try to reach for my phone on the table, I sometimes suffer but now, I can easily pick it up.’ (Participant 5, male, transport officer)

## Discussion

Rehabilitation helps stroke survivors to return to their community life after stroke and to be functionally independent. The authors aimed to explore and describe the views of stroke survivors on the impact of rehabilitation on their community life. The results show an overall improvement in the community life of participants following rehabilitation. However, most participants reported difficulty with social or leisure activities as well as an inability to return to work.

All participants mentioned being active and independent in performing their activities of daily living prior to the stroke. However, stroke changed their lives. Stroke affected their functional independence as they were unable to perform their activities of daily living, which included self-care or personal activities, home or family activities, work activities and social or leisure activities. The findings are similar to other studies (Cawood, Visagie & Mji 2016; Kalavina et al. 2019; Opoku, Eliason & Akpalu 2020), which report that stroke leads to limitations with performing activities of daily living and also interferes with social activities of the survivors. Most of the participants reported functional limitations post-stroke as they had to be assisted to perform their daily activities such as feeding, bathing, grooming, toileting, among others. As they had lost the ability to function, they could not go to work or enjoy social or leisure activities.

Most participants mentioned improvements in performing their self-care or personal activities following 6 months of rehabilitation. They are independent with bathing, grooming, continence, feeding, and the like. A study by Chinchai et al. (2020) reports that stroke survivors have improvements in carrying out their activities of daily living including self-care following rehabilitation. However, participants reported on their inability to perform their home or family duties, resume work, or enjoy social or leisure activities independently for various reasons. Most had not regained the full function of the affected upper limb, and this affected their ability to function independently. Stroke survivors have a slower recovery of hand function (Borschmann & Hayward 2020). This could be a possible reason why most participants reported not being able to independently perform their household activities.

Most participants had not been able to return to work because of the presence of residual impairments with mobility and hand function. Kalavina et al. (2019) also report that most stroke survivors are unable to return to work despite undergoing rehabilitation. Some of the participants mentioned being easily tired with little activity and as such, they had not been able to return to work. Stroke survivors therefore need some form of vocational retraining to help them to return to work despite the residual impairment. Also, to get stroke survivors to return to work with their residual impairment, there is the need to provide the necessary environment suitable for them based on their impairment as well as provide them with the necessary social support (Balasooriya-Smeekens et al. 2016). This means that there should be a vocational or occupational aspect to stroke rehabilitation to help get stroke survivors return to work. Lindgren et al. (2022) in Sweden found that most stroke survivors report being able to return to work after stroke and they were happy with going back to work despite their residual disability. The stroke survivors were able to return to work after stroke because they had received the necessary social support at their workplaces and they also had supportive working environments (Lindgren et al. 2022). This is in contrast to our findings. Attention needs to be given to the occupational aspect of rehabilitation as this will help stroke survivors to find adaptive ways of independently carrying out their home or family roles as well as return to work.

Although participants had improved functionally, they were not interested in social or leisure activities and they tended to isolate themselves from people as has been reported in other studies (Bhogal et al. 2016; Kalavina et al. 2019). Bhogal et al. (2016) discussed the possible reason for stroke survivors ignoring social activities despite their physical improvement, finding that stroke survivors lack the required skills to navigate through their leisure or social activities in the presence of their disability. Also, most stroke survivors are unable to enjoy social or leisure activities because of the perception that others would be watching them and gossip about their condition as we report. Kalavina et al. (2019) reported that stroke survivors isolate themselves from social activities because they are usually sidelined and face social stigmatisation. Therefore, there is the need to incorporate social and psychological support in the rehabilitation of stroke survivors. The authors recommend the establishment of social support groups for stroke survivors in Ghana to help deal with the issue of isolation and stigmatisation as well as enhancing social engagement. This will also create awareness for the need for stroke survivors to move out and enjoy social or leisure activities. Stroke survivors will therefore have some sense of acceptance of the perceived changes in their body image. This will also help to deal with the issue of boredom, which can lead to depression and depression can also affect the ability of the stroke survivor to recover well functionally (Butsing et al. 2019).

All participants reported receiving only physiotherapy rehabilitation in addition to their medical check-ups. In Africa the most available rehabilitation services for stroke are medical and physiotherapy services (Urimubenshi et al. 2018). This could probably explain why stroke survivors were unable to go to work as well as enjoy social or leisure activities as the occupational and psychological parts of rehabilitation are limited.

### Limitations

The authors used a qualitative method that makes generalisability of the obtained data limited. The data obtained are limited to the settings where data were collected. The authors therefore recommend conducting similar studies in other settings across the country in order to have more data across the country on the impact of rehabilitation on stroke survivors.

## Conclusion

The authors aimed to investigate the impact of rehabilitation on stroke survivors in the Greater Accra Region of Ghana. Although most stroke survivors mentioned having some functional improvement following 6 months of rehabilitation, they were unable to return to work and enjoy social or leisure activities. This shows that attention needs to be given to the activity and participation aspects of functioning in addition to the physical aspect. Stroke rehabilitation must be complete, involving physical, social, occupational, and psychological management to enable stroke survivors to get an all-round improvement in function. When rehabilitation is complete, stroke survivors may be able to return to their community life, perform all their functional tasks, return to work and also enjoy social or leisure activities.
